# Analysis of the following Related Surgeries of Unicompartmental Knee Arthroplasty and Total Knee Arthroplasty: A Taiwanese National Health Insurance Research Database Population-Based Study

**DOI:** 10.1155/2020/9713726

**Published:** 2020-10-16

**Authors:** Shu-Hao Chang, Chune-Chen Lee, Chia-Ying Lin, Yu-Feng Kuo, Ching-Chuan Jiang, Yong-Chen Chen

**Affiliations:** ^1^Department of Orthopedics, Fu Jen Catholic University Hospital, Fu Jen Catholic University, No. 69, Guizi Rd., Taishan Dist., New Taipei City 24352, Taiwan; ^2^School of Medicine, College of Medicine, Fu Jen Catholic University, No. 510, Zhongzheng Rd., Xinzhuang Dist., New Taipei City 24205, Taiwan; ^3^Master Program of Big Data in Biomedicine, College of Medicine, Fu Jen Catholic University, No. 510, Zhongzheng Rd., Xinzhuang Dist., New Taipei City 24205, Taiwan

## Abstract

**Background:**

Current treatment options for both unicompartmental knee arthroplasty (UKA) and total knee arthroplasty (TKA) are still controversial with no consistent results in which one is superior to others. This is the first study to examine and analyze the following related data available in patients receiving either UKA or TKA from the National Health Research Database (NHIRD) in Taiwan.

**Methods:**

The database was searched from NHIRD, pooling one million random patients. Patients' age, gender, and comorbidities were analyzed in either UKA or TKA between January 2005 and December 2013, or up until death. For the patients that had received bilateral surgeries, further subgrouping was divided into TKA to TKA, UKA to UKA, TKA to UKA, and UKA to TKA to analyze the completion rate curve. Additional analysis of the order codes 64202B, 64053B, and 64198B was defined as failures, and the related failure rate curves were analyzed separately within ten years. Finally, infection-related codes were analyzed.

**Results:**

6,179 patients (*n* = 276 UKA; *n* = 5903 TKA) were selected. Age (*p* < 0.0001) and gender (*p* = 0.037) had significant differences, with more young population and males having UKA than TKA. Most comorbidities had no significant difference. For the bilateral surgery analysis, the UKA to UKA group had the fastest completion rate (*p* < 0.001) and UKA to TKA was the slowest. There were no significant differences in the failure rates of 64202B, 64053B, and 64198B.

**Conclusion:**

Most UKA and TKA are appropriate solutions to treat patients with osteoarthritis or osteonecrosis. UKA to UKA is the quickest bilateral completion surgery, and UKA has a higher chance of undergoing revision surgery than TKA.

## 1. Background

Severe stages of osteoarthritis (OA) and osteonecrosis (ON) often lead to hip or knee arthroplasty [1]. In Taiwan, the current prevalence of OA among the elderly population over 50 years old is approximately 37% [2], in which this statistic will likely continue to increase. Currently, ON is one of the leading indications for primary total hip arthroplasty (THA) accounting for 46.9% [3]. Both OA and ON consist of several noninvasive treatments implanted at individual protocol including weight loss, activity modification, physical therapy, and nonsteroidal anti-inflammatory drugs (NSAIDs) [4]. If symptoms continue, surgical procedures such as high tibial osteotomy (HTO) or knee arthroplasty are necessary to provide symptomatic relief and to promote joint function [5]. Primary knee arthroplasty is commonly recommended where it is categorized into two types: unicompartmental knee arthroplasty (UKA; replaces only the affected knee compartment) and total knee arthroplasty (TKA; replaces the entire knee joint) [6]. Although there are several controversies regarding the use of UKA, such as being associated with a higher risk of revision compared with TKA, UKA nevertheless has shown to be a good treatment option [6–8]. Previous studies have claimed UKA had advantages such as less rehabilitation time requirements, preservation of soft tissues and bone stocks, better functional outcome and range of motion, subjective preference, and less pain in comparison to TKA [6–10].

In Taiwan, previous studies were conducted using Taiwan's national database focused more on TKA, such as the cost comparison between staged versus simultaneous bilateral TKA [11] and a 15-year retrospective study in cancer patients [2] analyzing mortality and periprosthetic infection rates after TKA [12]. Therefore, to the best of our knowledge, this study will be the first to examine the following related analyses of surgeries from patients receiving UKA and TKA in Taiwan. The aim is to examine (1) the characteristics of the patients, (2) the completion rates for bilateral surgery in groups from TKA or UKA to the following UKA or TKA within eight years, and (3) the additional subgrouping of UKA and TKA revision surgery comparison using the order codes of 64202B, 64053B, and 64198B.

## 2. Methods

### 2.1. Data Source and Ethical Considerations

Datasets were retrieved from the National Health Insurance Research Database (NHIRD) in Taiwan, which represents most population, if not all, as it covers over 99% of the Taiwan population (approximately 23 million residents) [11]. By providing abundant information from ambulatory and inpatient care to prescription and medication data, the NHIRD had been commonly used by many researchers for hundreds of published studies [13]. Due to the research cost restrictions, the patient sampling data that were collected in this study constitute the Longitudinal Health Insurance Database (LHID) 2005. The entire original claimed data of one million beneficiaries enrolled in the year 2005 was randomly sampled from the year 2005 Registry for Beneficiaries of the NHIRD, who was a beneficiary of the NHIRD program from January 2005 to January 2006. There will be no significant difference in the gender distribution between the patients in the LHID 2005 and the original NHIRD.

### 2.2. Study Population and Procedure

**Figure 1:**
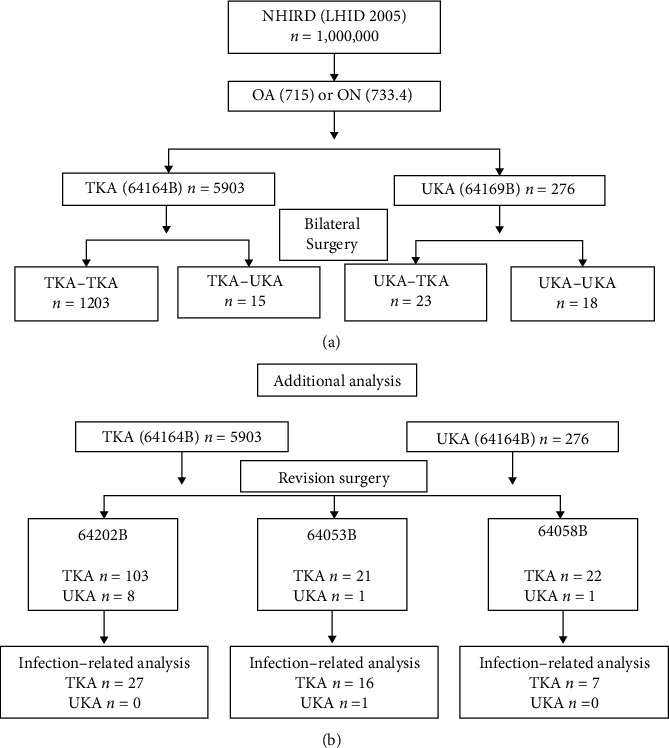
Flow chart of the database analysis: (a) primary analysis of bilateral knee arthroplasty; (b) additional analysis of revision surgery and infection-related analysis. Abbreviations: NHIRD: National Health Insurance Research Database; LHID: Longitudinal Health Insurance Database; OA: osteoarthritis; ON: osteonecrosis; UKA: unicompartmental knee arthroplasty; TKA: total knee arthroplasty.

This study is a population-based database cohort study, analyzing patients receiving UKA or TKA between January 2005 and December 2013, or up until death.  Figure 1(a) shows the flow chart of the analysis. Patients that were diagnosed and defined by the recording of the International Classification of Diseases, Ninth Revision, Clinical Modification (ICD-9-CM) Code 715 for OA and Code 733.4 for ON were included. If patients had received a previous arthroplasty before the index date, they were excluded to avoid confounding effects. After defining the diagnosis of patients in either OA or ON, based on when the patients have been admitted to the hospital, additional diagnosis of two surgery order codes in the NHIRD data, 64164B (TKA) or 64169B (UKA), was added. Baseline characteristics of each patient's age, gender, and comorbidities and complications (myocardial infarction, congestive heart failure, peripheral vascular disease, cerebrovascular disease, dementia, chronic pulmonary disease, rheumatic disease, liver disease, diabetes mellitus, etc.) before or at time of the index date based on the Charlson Comorbidity Index (CCI) [14] were collected and analyzed. After identifying patients with TKA and UKA, additional description of patients receiving bilateral knee arthroplasty at different time points was then divided into four groups: TKA to TKA, TKA to UKA, UKA to TKA, and UKA to UKA. The completion rate was analyzed at this point.

Additional analysis is shown in Figure 1(b). Patients with TKA and UKA were divided into three NHIRD surgery order codes focusing more on revision surgery: Code 64202B (revision total knee replacement), Code 64053B (arthrotomy for the acute septic joint), and Code 64198B (removal of a prosthesis). Code 64202B meant the patients' first artificial joint had to be replaced for any reason after their failure. Two other codes that represent the artificial joint may need to be debrided or removed due to a possible infection. Lastly, infection-related codes were analyzed in each revision surgery codes, to find out what other related diagnosis or reason was made during the period between a year before the revision surgery and two weeks after their revision surgery. ICD-9-CM Codes 711.0 (pyogenic arthritis), 711.06 (pyogenic arthritis involving lower leg), 711.9 (unspecified infective arthritis), 711.96 (unspecified infective arthritis in the lower leg), 996.66 (infection and inflammatory reaction due to the internal joint prosthesis), and 996.67 (infection and inflammatory reaction due to another internal orthopedic device, implant, and/or graft) were analyzed. Additional ICD-9-CM Codes 733.81 (malunion of fracture), 996.41 (mechanical loosening of the prosthetic joint), 996.42 (dislocation of the prosthetic joint), 996.43 (prosthetic implant failure), and 996.44 (periprosthetic fracture around the prosthetic joint) were included as well.

### 2.3. Data Collection

All characteristics of the patients were available from the database. The index date was set prior to the day each patient received their first completion of UKA or TKA recorded by the administrative insurance database. The end of the follow-up was death or until December 31, 2013.

### 2.4. Statistical Analysis

This study utilizes SAS (SAS Institute, Cary, NC, USA) for data integration and statistical analysis. A two-tailed test of *p* value < 0.05 was considered statistically significant. Descriptive statistics were performed for demographic characteristics (age, gender, comorbidities, and complications). For assessing the risks of receiving another arthroplasty surgery in the future, a 95% confidence interval was used to express the correlation and statistically significant difference. In analyzing the failure rate from patients' first surgery to their follow-up surgery, the Kaplan-Meier estimator was used. The log-rank test was used to find the differences in failure rate and completion rate.

## 3. Results

Figures 1(a) and 1(b) display the total number of patients that were included and analyzed.  Table 1 shows the baseline characteristics of 276 UKA patients and 5903 TKA patients. Both age (*p* < 0.0001) and gender (*p* = 0.037) showed significant differences from the total number of patients that underwent TKA and UKA. In patients that were 60 years old or older, 73% of patients had UKA while almost 90% of patients had TKA. Both groups had more females than males; however, the ratio of males in UKA was higher than TKA. Lastly, in comorbidities and complications, the peripheral vascular disease had a significant difference (*p* = 0.045) while the others had no significant differences. Diabetes mellitus (type I and II) had the highest proportion out of the other categories where 14.8% of the UKA patients and 17.7% of the TKA patients were diabetic.

**Figure 2:**
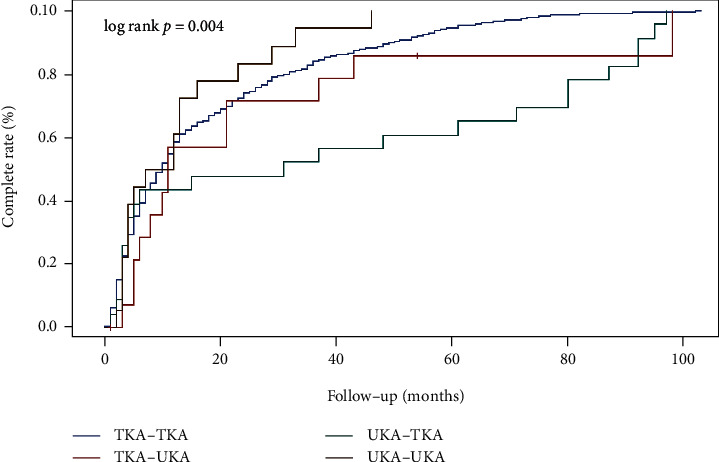
Complete conversion rate comparing patients' first surgery following their overall surgery within an 8-year span.

Table 2 with Figure 2 analyzed further the completion rate of bilateral surgeries in patients who underwent either TKA or UKA followed by another TKA or UKA on their contralateral side. UKA followed by UKA had the least number of patients along with the quickest completion of up to 40 months in comparison to TKA followed by TKA having the most number of patients with the longest completion. There was a significant difference in the curves (log-rank test; *p* = 0.0004) in Figure 1.

**Figure 3:**
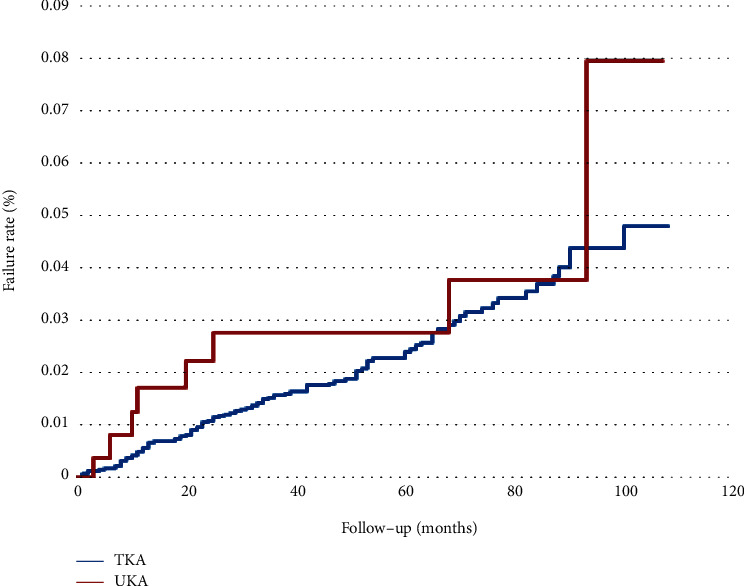
Failure rate comparing UKA and TKA in revision total knee replacement only (64202B) within a 10-year span.

**Figure 4:**
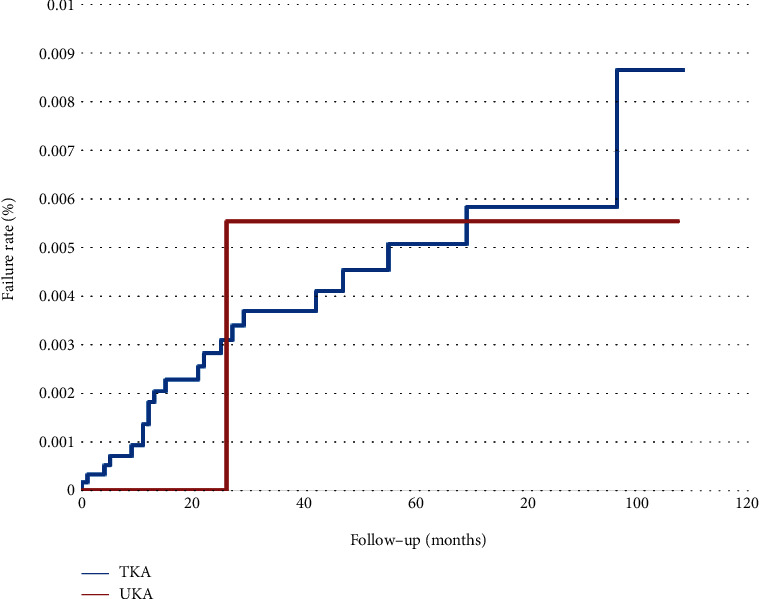
Failure rate comparing UKA and TKA in arthrotomy for acute septic joint only (64053B) within a 10-year span.

**Figure 5:**
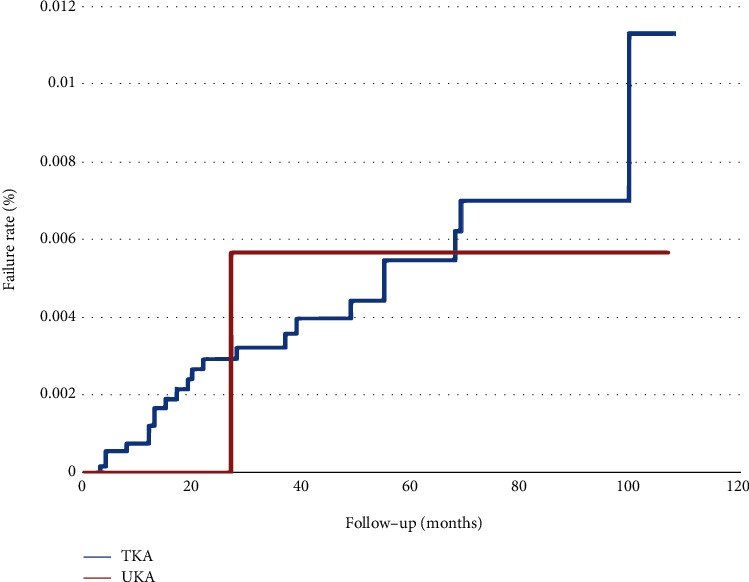
Failure rate comparing UKA and TKA in the removal of prosthesis only (64198B) within a 10-year span.

Figures 3–5 analyze the subgroups of surgery order codes including 64202B, 64053B, and 64198B of UKA or TKA. In Figure 3, 103 patients in TKA and 8 patients in UKA failed. UKA had a higher failure rate than TKA, with a failure rate of TKA and UKA, 1.74% (100%-98.26%) and 2.9% (100%–97.1%), respectively. However, there was no significant difference in the failure rate curve between the two groups (log-rank test; *p* = 0.3382). In Figure 4, 21 patients in TKA and 1 patient in UKA failed. The failure rates for both were 0.36% with no significant difference between the two groups (log-rank test; *p* = 0.9586). In Figure 5, 22 patients in TKA and 1 patient in UKA failed. The failure rates for TKA and UKA are 0.37% and 0.36%, respectively, with no significant differences (log-rank test; *p* = 0.8969).

Table 3 shows the infection-related codes to 64202B, 64053B, and 64198B of UKA and TKA. Most revision surgeries were undergone because of Codes 711.0 and 711.9. None of the other ICD-9-CM: 733.81, 996.41, 996.42, 996.43, 996.44, 996.66, and 996.67, were found.

## 4. Discussion

The current treatment options for knee arthroplasty are still controversial where both UKA and TKA are both utilized to treat OA and ON. No consistent results have been shown in previous literatures; however, the use of both UKA and TKA has been increasing worldwide [15]. To our knowledge, this is the first study to examine the national database of patients that have undergone either UKA or TKA from 2005 to 2013 in Taiwan.

Our study in Table 1 has found patients who have received TKA or UKA that were most likely 60 years and older. Taiwan has been defined as an aging society where the population over 65 years old has reached 7% in total since 1993 [16]. By 2025, the percentage of the Taiwan population that is over 65 years old is forecast to increase up by 20% [17]. Some surgeons still might regard UKA as a temporary procedure and recommend that patients over 60 years of age are still best treated with TKA [15]. Our research has shown that the age distribution of patients receiving UKA is younger than in TKA. UKA may possibly be used to the affected knee compartment in younger patients who had OA from possible joint injury or repetitive joint stress in overuse. TKA was probably recommended to replace the entire knee joint in older patients who has OA or ON from aging.

In this study, distribution in females was more than males in both groups but the ratio of males in UKA was more. This could possibly be explained by more male workers that were younger than in TKA. Lin and colleagues showed a similar trend from their previous study of a 15-year retrospective study in Taiwan [2]. Their demographic trend also showed the rates increased were much higher in women than in men, where men were increased by 2.7 times, from 13.56 to 37.09 per 100,000, and women were increased 3.1 times, from 36.35 to 112.36 per 100,000 [2].

For comorbidities and complications, hip and knee replacements were common among diabetic patients. Even if there were no significant differences (*p* = 0.219) in our study between TKA and UKA, the total numbers for diabetic patients (TKA *n* = 1047, UKA = *n* = 41) were more than other comorbidities and complications. In the United States, a 15-year study claimed a total of 8.55% diabetic patients from a total of 750,000 joint replacement patients [18] that have a high prevalence in undergoing total joint arthroplasty [19]. From our database in Taiwan, the ratio of diabetes mellitus is even higher, with 14.8% in UKA and 17.7% in TKA, With THA and TKA patients, diabetes mellitus influences functional and perioperative clinical outcomes. For example, if arthroplasty patients are insulin-dependent diabetes, they are more likely to experience medical complications and readmitted within 30 days of surgery [20].

Previous studies have shown several revision rate percentages; however, what was often seen was the UKA to TKA revisions that were done frequently [21–23]. UKA has shown to be a good treatment as its advantages over TKA consists of preservation of soft tissues and bone stock, better functional outcome, and less need for blood transfusion in the immediate postoperative period [7]. However, the most common failure modes for UKA are instability, the progression of diseases from another compartment, and the aseptic loosening of the tibial component [21, 22] whereas TKA to TKA were often rerevised from deep infection. Leta and colleagues [23] analyzed UKA to TKA (*n* = 578) and TKA to TKA (*n* = 768) using their national registry database in Norway from 1994 to 2011. Overall, with a rate of rerevision from UKA to TKA of 12% and TKA to TKA of 13%, they were both comparable with a ten-year survival rate of 82% and 81%, respectively. In conclusion, they claimed that both UKA to TKA and TKA to TKA had similar outcomes in terms of survival, functional outcome, level of pain, satisfaction in patients, and changes in health-related quality of life. In another study using the Australian Orthopaedic Association National Joint Replacement Registry from 1999 to 2008 [24], they claimed a similar conclusion that even if UKA to TKA rerevision still achieves the best outcomes, there are risks to keep in mind with similar risk in primary TKA.

In our study, the completion rate of UKA to UKA is faster than other completion groups. If UKA to UKA was mostly contralateral surgeries, this could be explained that UKA had a faster recovery rate, less tissue damage, lower pain, and faster recovery time than TKA [24]. If UKA to UKA was mostly ipsilateral of the same side, this type of revision may be rare and could not possibly happen because of the higher risk of rerevision than the conversion of a UKA to TKA [21]. Both TKA to TKA and TKA to UKA were slower than UKA to UKA. This could be explained by the slower recovery time and the increase in soft tissue destruction. Lastly, UKA to TKA took the longest time to complete. From the conservative initial procedure to the primitive mode of failures to its consequences on bone stock and ligament integrity, conversion of UKA to TKA can be complicated than a primary TKA [25]. After the initial UKA, the possibilities the contralateral side may perhaps have started to degenerate were initially not as problematic until time gradually passed.

There are some possible scenarios with the recommended revision of UKA, such as an early case of aseptic loosening of a single component, liner change in case of mobile-bearing dislocation with a revision to a thicker polyethylene, isolated polyethylene wear when diagnosed early, revised before metal-on-metal wear or osteolysis. The recommended revision of TKA is commonly known for its deep infection. Therefore, subgrouping of revision of total knee replacement, arthrotomy for acute septic joint, and removal of the prosthesis were necessary to further analyze its failure rate within the eight years, analyzing the possible revision surgery for not only UKA but also TKA as well.

UKA has a higher failure rate than TKA in this study, with a failure rate of TKA and UKA, 1.74% and 2.9%, respectively. This could be explained by the continuous cartilage degeneration from the remaining cartilage after UKA was completed. In patients that had done TKA, the whole cartilage had been replaced with artificial and no further cartilage wear would appear. Therefore, the implant survival rate of TKA was higher than UKA. Dyrhovden et al. [26] also concluded that the survival rate had improved for TKA during a ten-year span, unlike UKA where it dramatically decreased. OA progression in knee compartments and aseptic loosening of components were a high risk and a frequent cause of UKA failure.

This study had two main limitations, due to the funding that used one million random sampling database LHID 2005. With the small number of samples of the UKA patients in comparison to a few thousands of TKA patients, this may affect the statistical analysis power. In suggestion to future research with additional funding, more researchers should enter the Ministry of Health and Welfare database to find out the true sample size comparison between UKA and TKA. In addition, even though our study had further analyzed the revision surgery, the patient's following related surgery may be the ipsilateral or contralateral knee. However, due to limited data not available in the database, this cannot be further analyzed.

## 5. Conclusion

To our knowledge, this is the first population-based database cohort study that reports the following related surgeries of UKA and TKA in Taiwan. UKA to UKA is the quickest bilateral completion surgery while UKA has a higher chance of undergoing revision surgery than TKA. While there is no consensus when it comes to comparing UKA and TKA, both have their own inherent complications and compensations with their ultimate goal of improved longevity and optimal function to treat patients with osteoarthritis or osteonecrosis.

## Figures and Tables

**Table 1:** Baseline characteristics of UKA and TKA patients.

	UKA (*N* = 276)		TKA (*N* = 5903)		*p* value
	*N*	%	*N*	%	
Age					<0.0001^∗^
<40	2	0.7%	11	0.2%	
40~49	13	4.7%	57	1.0%	
50~59	58	21.0%	524	8.9%	
60~69	89	32.2%	2026	34.3%	
>70	114	41.3%	3285	55.6%	
Gender					0.037^∗^
Female	189	68.5%	4376	74.1%	
Male	87	31.5%	1527	25.9%	
Comorbidities/complications					
Myocardial infarction	1	0.4%	9	0.2%	0.367
Congestive heart failure	0	0.0%	47	0.8%	0.272
Peripheral vascular disease	1	0.4%	0	0.0%	0.045^∗^
Cerebrovascular disease	1	0.4%	71	1.2%	0.379
Dementia	2	0.7%	10	0.2%	0.098
Chronic pulmonary disease	7	2.5%	131	2.2%	0.728
Connective tissue disease	1	0.4%	44	0.7%	0.722
Ulcer disease	6	2.2%	132	2.2%	1.000
Mild live disease	1	0.4%	27	0.5%	1.000
Diabetes mellitus (type I and II)	41	14.8%	1047	17.7%	0.219
Diabetes associated with end organ damage	0	0.0%	10	0.2%	1.000
Hemiplegia	1	0.4%	2	0.0%	0.128
Chronic kidney disease/chronic renal failure	2	0.7%	34	0.6%	0.675
Malignant tumor	3	1.1%	26	0.4%	0.138
Moderate to severe liver disease	0	0.0%	1	0.0%	1.000
Metastatic tumor/cancer	0	0.0%	3	0.1%	1.000
Acquired immune deficiency syndrome	0	0.0%	0	0.0%	—

Abbreviations: UKA: unicompartmental knee arthroplasty; TKA: total knee arthroplasty. ^∗^Significant difference.

**Table 2:** Total patient's complete conversion rate span between their first surgery and their following surgery within 10 years.

	1^st^ month	20 months	40 months	60 months	80 months	100 months
UKA-UKA	18	4	1	0	—	—
UKA-TKA	23	12	10	9	7	0
TKA-UKA	15	6	3	1	1	0
TKA-TKA	1203	386	170	67	13	3

Abbreviations: UKA: unicompartmental knee arthroplasty; TKA: total knee arthroplasty.

**Table 3:** Total number of patients from revision surgery analysis in infection-related surgery codes.

Infection-related codes	TKA			UKA		
	64202B	64053B	64198B	64202B	64053B	64198B
None	76	5	15	8	0	1
711.0/711.9^∗^	2	3	1	0	0	0
711.0	21	13	6	0	1	0
711.9	4	0	0	0	0	0
733.81	0	0	0	0	0	0
996.41	0	0	0	0	0	0
996.42	0	0	0	0	0	0
996.43	0	0	0	0	0	0
996.44	0	0	0	0	0	0
996.66	0	0	0	0	0	0
996.67	0	0	0	0	0	0
Total	103	21	22	8	1	1

Abbreviations: UKA: unicompartmental knee arthroplasty; TKA: total knee arthroplasty. ^∗^Combination of 711.0 and 711.9.

## Data Availability

These data were available to us as staffs of the Department of Orthopedics at Fu Jen Catholic University Hospital and at Fu Jen Catholic University, using the National Hospital Research Database (NHIRD) with using a specific data subset for research purposes called Longitudinal Health Insurance Database (LHID) 2005. These data are protected by the Ministry of Health and Welfare and patient privacy laws in Taiwan; no public links are available to these protected health information datasets. These data will be made available to others after appropriate data privacy and human subject approvals needed by the institution. Requests should be sent to aberchangtw@gmail.com.
